# Senomic view of the cell: Senome *versus* Genome

**DOI:** 10.1080/19420889.2018.1489184

**Published:** 2018-08-10

**Authors:** František Baluška, William B. Miller, Jr

**Affiliations:** a IZMB, University of Bonn, Bonn, Germany; b Paradise Valley, USA

**Keywords:** Cell, cognition, communication, Genome, Epigenome, evolution

## Abstract

In the legacy of Thomas Henry Huxley, and his ‘epigenetic’ philosophy of biology, cells are proposed to represent a trinity of three memory-storing media: Senome, Epigenome, and Genome that together comprise a cell-wide informational architecture. Our current preferential focus on the Genome needs to be complemented by a similar focus on the Epigenome and a here proposed Senome, representing the sum of all the sensory experiences of the cognitive cell and its sensing apparatus. Only then will biology be in a position to embrace the whole complexity of the eukaryotic cell, understanding its true nature which allows the communicative assembly of cells in the form of sentient multicellular organisms.

## Introduction

Since the discovery of DNA and its role in coding proteins some sixty years ago, the biological sciences have accomplished a breakthrough revolution, evolving from an imprecise, descriptive science of associations into its more mature status that can stand in equivalent juxtaposition to the previously dominating fields of physics and chemistry. However, in order to complete this revolution, something fundamental is still missing from modern biology, as evidenced by difficulties in tackling the “hard questions” such as biological nature of feelings, emotions, qualia (subjective sum of all sensory experiences) and consciousness. This is not any trivial problem. Illumination of these phenomena is crucial for our understanding of our human nature as well as the world around us, both its biotic and abiotic parts.

In the legacy of Thomas Henry Huxley [1], cells are proposed to represent a trinity of three memory-storing media. Upstream of the genome and epigenome, the bioelectric senome acts in close association with the plasma membrane. Recent advances in neurosciences make it clear that feelings, emotions, qualia and consciousness are very real biological phenomena based on chemistry and fundamental biology [2–7]. Sleep, qualia, consciousness and cognition, all appear to represent essential living qualities, not only for humans, but for all living organisms as they struggle to survive in a hostile environment [8–17]. However, the true nature of these inherent phenomena is still elusive and highly controversial.

If feelings, emotions, cognition, qualia and consciousness are based on neurochemical toolkits and processes [2,3,7,9,18–21], their true nature should be decipherable. Nevertheless, we obviously fail in this crucial aim, suggesting that our current approach is missing some essential aspects of these phenomena and/or our approaches and methodology are flawed. Here we suggest that in order to succeed in resolving the biological basis of these fundamental aspects of living organisms, we need to add a new science to our biological agenda that focuses on sensory biology linked to cellular cognition and behavior. This new science of feelings, emotions, qualia and consciousness must be able to tackle these issues from an evolutionary perspective [4–7,16,18–22]. Its further requirement is an explanation of the emergence of a variety of elusive biological phenomena in their hypothetical ancient proto-states at the level of the simplest unicellular organisms [7,14,16,22,23], such as proto-feelings, proto-emotions, proto-qualia, proto-consciousness [7,24] which could then be further engaged as complexation in multicellular organisms.

**Box 1.** Components and bioelectric field-like nature of *Senome* with implications for multicellularity and social cognition.

It is well understood that every atom generates its own electromagnetic field (EMF). For example, Magnetic Resonance Imaging is dependent on that phenomenon. Every living organism is dependent on this electrical circumstance and the resultant electrostatic attractional forces that enable the atomic bonding that generates every type of matter. In biological membranes, especially the plasma membrane, ion channel and transport activities generate bioelectric fields [116,117] which also summate to generate cell, tissue and organ-specific fields. These act as coherent resonances that assist in the integration and organization of multicellular organisms such as plants, fungi, animals and humans. For example, self-generated bioelectic fields were reported around apices of tip-growing pollen tubes, root hairs as well as whole roots [118–125]. Similarly, cells of all organisms are known to generate bioelectric fields and also to sensitively respond to such bioelectric fields [126–130]. Especially prominent bioelectric fields are generated by neurons, generating feedback loops that synchronize whole neural networks [131–135]. Such strong bioelecric fields extend around brains and can be routinely reorder as local field potentials (LFPs) that can be measured on electroencephalograms (EEGs) [126,136,137]. All such electrodynamic fields, even biophotonic ones, function as trans-membrane potentials that are crucial for sperm activation, embryonic development, cell migration, stem cell differentiation, cell regeneration and gene expression in all complex multicellular organisms[138]. Beyond internal cellular dynamics and cell-cell communication, many fields, such as EMFs, can be crucial external sources of cell stimulation at the macro-organic level. Migrating birds, hunting eels and sharks or flowers attracting their insect pollinators: all use their own electric fields [139–146]. Behavioral and cognitive effects in humans have been documented too. Electric stimulations are used for the treatment of depression, bipolar mood disorders and mood elevation [147,148]. For the molecular composition of signal transduction and information-processing networks of the *Senome*, see references 56, 63, 116, 117, 126–137.

## The cell in the 21st century

Recently it is emerging that all cells [25] demonstrate basal cognition [8,14,16,23,26–33]. Indeed, it has been argued that life should properly be defined through the property of self-referential cognition [31,34]. Furthermore, recent advances have also revealed that neuronal aspects and cognitive capacities are valid concepts for unicellular organisms too [14,16,22,35]. Even bacteria show sensory complexity and sensory-motor circuits between plasma membrane-based sensors and correspondent motor responses of their flagella modulated by previous experiences [14,36]. It has also been demonstrated that plants have complex sensory systems which feed into their adaptive behavior [37–39]. Tropisms of plant organs, such as gravitropism and phototropism, are based on cellular-based sensory events that are spatially separated from motoric zones [37,39,40]. Rapid electrical and slower chemical cell-cell communications, accomplished across plant synapses, are inter-linking and integrate sensory-motoric circuits of plant organs. Although these plant synapses differ in their molecular toolkit and some structural aspects from the animal neuronal synapses, they are organized through very similar principles and also perform analogous tasks [39–46]. Furthermore, neuronal synapses have deep evolutionary origins and have evolved from proto-synapses [9,47] which might underlie primordial sentience based on proto-feelings, proto-emotions, proto-qualia, and proto-consciousness [7,24]. It can be argued that such a deep evolutionary origin of neurons and synapses implies that these derivative qualities are inherent aspects of cellular organisms which are as fundamental to unicellular existence as they are known to be for multicellular organisms [8,30,43,48].

All biological organisms during their evolution are shaped by their self-referential adaptation to an ever-changing environment [31,49]. As this environment is often rather hostile and extremely variable, all organisms in order to survive need to extract, process, store and use faithful and effective information about their environment [49,50]. It has been previously argued that this process of information extraction and reception, and its further communication and deployment, is based on a consistent reiterative self-referential information cycle that undergirds all cellular life [31,34,49–51]. In such circumstances, genetic information and epigenetic inputs must be viewed as elements of a toolkit as part of the cell-wide information cycle by which cellular organism sense and adapt to environmental stresses [51]. As a derivative then, information which had been encoded in DNA sequences of any ancient organisms was first perceived as sensory information. This means that sensory information is upstream of DNA information. Since there is no indication that DNA directly participates in the acquisition of sensory data, its use as memory and as a reproductive tool is a dependent function of an entire cellular sensory architecture by which environmental cues can be assessed and deployed. As this is information-dependent action, the cell is best understood as a form of informational architecture which is heavily dependent on sensory informational cues received from the external environment [49,51,52].

It is therefore proposed that insofar as DNA sequence coding for a particular product is termed the *gene*, each particular sensory experience subjectively perceived by any organism should be termed the *sene*. In order to survive, organisms must retrieve as much information as possible in form of the senes that relates to the true nature of their environment. It could be imagined that those organisms which were not able to retrieve senes that truly corresponded to their environment were weeded-out via filtering Darwinian selection. In this process, the complexity and fidelity of the senome continuously increased during biological evolution. This senomic information is essential for any organism, not only with respect to its adaptive behavior (*akin* software), but also as safe storage of their most relevant aspects within their genomes (*akin* hardware). Together, these constitute the informational architecture of the living cell as essential elements of cell-wide cognitive information management system. It is this dynamical coupling that unites the senome with genome to critically intersect with the variety of memory-encoding mechanisms that characterize the fully functioning cell. It is argued that in order to unravel this inherent duality of dynamic interactions between sensory and DNA/RNA-based information, the biological sciences must put more efforts on sensory aspects of biology as an integration in which sensory information and memory are both sides of the “same coin” that can instill a new biological sciences of the new 21st Century.

## Senome of cellular life

Therefore, in accordance with the genetic sciences, in which the sum of all genes is termed the *Genome* and that science which aims at illuminating all processes related to the genome is called *Genomics*; the sum of all *Senes* should be termed the *Senome* and the science behind it, *Senomics*. Only such a focus and approach to these self-referential phenomena will allow us to fully understand their biological basis as well as their true non-genomic and primary nature. Since any viewpoint that the cell is a functional automaton no longer comports with scientific data, those sources of informational input and the complex mechanisms by which information is assessed, communicated, deployed and managed becomes the new ground state of any detailed understanding of cellular life or the holobionts that are its collective product [31,53]. As each biological organism is inevitably a subject, and its world-view is constructed internally from its particular self-referenced *Senome* evolved during its specific evolutionary trajectory and enfolded during its individual life, it is obvious that the *Senome o*f each organism is unique.

Moreover, dynamic environment–organismal interplay shapes *Senomes* of individual organisms during their life, so that even two genetic twin organisms will move apart with respect to their *Senomes*, according to their unique personal experiences which necessarily accumulate during their individual life histories. Importantly, although this has been conventionally acknowledged as a function of acquired epigenetic experiences, the *Senome* concept includes the memory encoded within the non-genomic structured arrangement of molecules [18–21,54] that also gets transferred through cell divisions or between generations [54]. Further too, the *Senome* includes the variety of sensory fields, such as electromagnetic, electrical, vibratory, or mechano-transduction, that store senomic information and transmit towards epigenome and genome (Box 1).

Clearly, replicating and cycling stores of information perpetuate life. It is argued that is the summation of all of these informational factors: *Genome, Epigenome*, and *Senome*. All these three systems, interwoven together, form the fully capable and adaptable cell. All three are also required participants in the complex interrelationships and communications that enable life and its survival.

In the case of unicellular organisms, the major organ for storing of genetic information is prokaryotic nucleoid or eukaryotic nucleus. On the other hand, the major organ for storing senomic information in all unicellular organisms is the plasma membrane, which represents the sensory boundary of the cell. This sensory boundary not only retrieves information from the environment but also displays this information akin to a holographic screen [55]. The plasma membrane is enriched with diverse sensors which are optimized during their evolution to retrieve faithfully information from the environment [56]. Selective and ordered activation of these sensors at the plasma membrane generates a 4-dimensional (spatial coordinates and time) senomic model of the outside world and assures its continuous updating [55]. In other words, the *Senome* is a smart perceptual “gate” which first receives and then channels all the acquired sensory information into the interior of the organism [55]. Here, this information is used to drive downstream metabolic and motoric processes according to the proscriptions of the cell’s information management system [31,34]. Therefore, it can be proposed that the *Senome* is the collective attachment to the informational matrix of the environment that propels internal cycles of biological information upon which all cells depend. Moreover, the emphasis of long-term information, which predominately proves to be relevant for organismal survival, is stored in the form of DNA sequences within the genome.

In the last ten years, epigenomics has emerged as an important counterpart of genomics in regulating biological organisms [57–60]. Epigenomics encompasses chemical modifications of DNA such as methylation, histones, chromatin-remodeling protein complexes [61], and other nuclear proteins, chaperones and some aspects of the dynamic cytoplasmic cytoskeleton [62]. All of these are deeply interlinked with the endocytic matrix [63] that integrates the Senome with the epigenome and genome [63].

**Figure 1.**
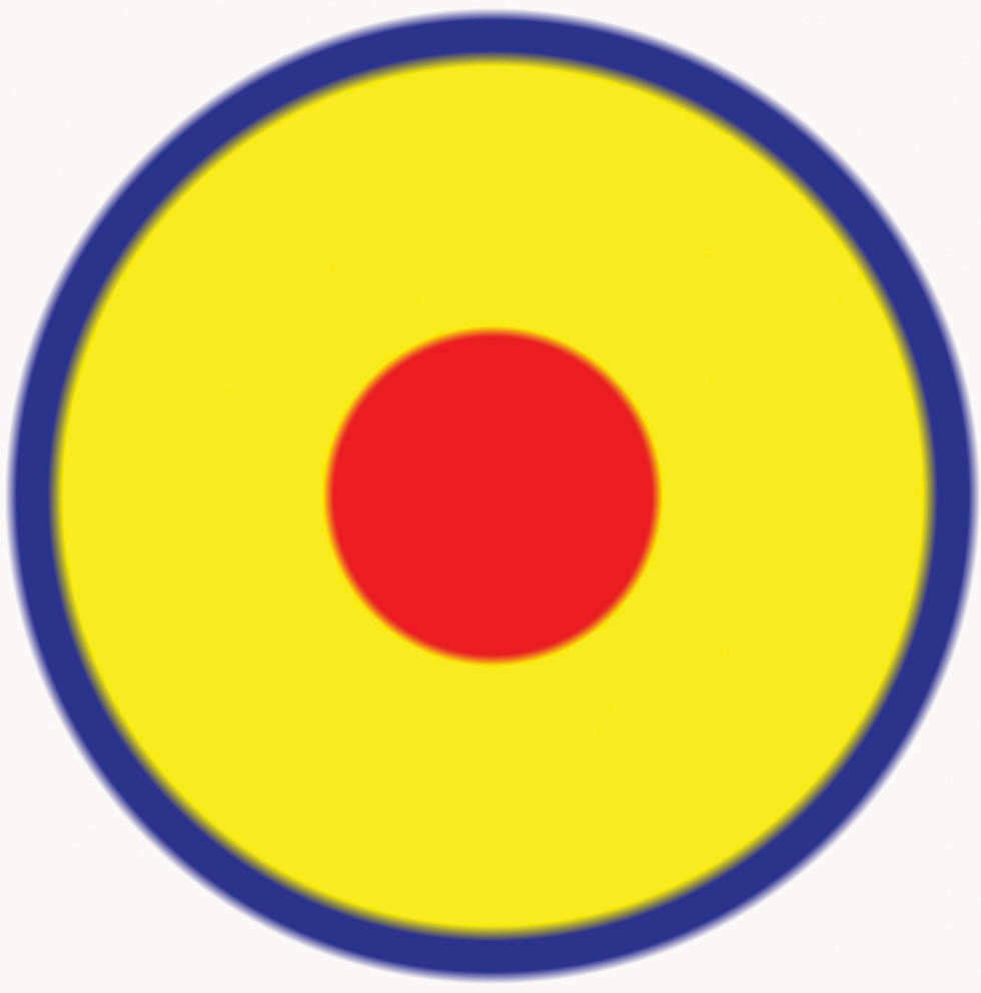
Senomic view of a cell. In this schematic and highly reduced view of a cell, the Senome is shown in blue, the Epigenome in yellow, and the Genome in red. The Senome encompasses the plasma membrane equipped with ion channels and transporters, vesicles and whole endocytic network. The epigenome encompasses all structures feeding from the Senome to the Genome, acting as a smart “translator” for dynamic feedbacks between the Senome and the Genome.

It is increasingly apparent that epigenomics is relevant for neuronal identity and plasticity, as well as mental states, disorders and behavior [57–59,64,65]. Certainly too, it is assumed that the genome of any organism has a similar over-arching function. Yet, neither the epigenome nor the intrinsic genome have provided satisfying answers to the complexity of human behavior (see the last section too). Therefore, it is proposed that in order to understand the cells that comprise any complex organism, such as ourselves, the addition of interactive senomics into our agenda is required. That *Senome* governs the primary aspect of living systems, encompassing both the epigenome and genome (Figure 1). Within the complex informational architecture of the cell, any environmental change first stimulates plasma membrane sensors which respond within seconds [56]. Only later, within minutes and hours, there might be downstream changes to the cytoskeleton, or other parts of the cell, such its epigenome. And, only then, will there be consequential expression from the genome.

## Implications of the cellular Senome

Our understanding of the cell and its intelligent adaptations will always remain incomplete if exclusively explored through epigenetics and genomics. When genes are properly assessed as among those tools that cells deploy in their extraction and use of information, then the need for the cell to have a sensing apparatus to respond to environmental stress becomes apparent. As an integral aspect of the cell, the senome comprises the summation of its active boundary conditions, including the plasma membrane amd the memory-encoded heritable molecular structural arrangements that particiate in the assessment, deployment and communication of information both within and without the cell.

All together, the Senome, Epigenome, and Genome form the entire informational architecture of the cell. It has been previously argued that the cellular form should be regarded as the biological expression of a self-referential information management system [34]. Within that self-referential cognitive frame, the Senome becomes a required link within any whole cell concept. It is the vital conduit of informational content and its transfer to the embedded systematic memory of the cell that maintains its homeostasic equipoise. It is well accepted that the cell attempts to sustain its most efficient homeostatic moment through the use and communication of self-referential information. Therefore, that process of sustaining homeostasis can no longer be viewed within a thermostatic model. Instead, it must be seen as a function of the active problem-solving capacity of any cell [34,66–71]. In such circumstances, a *Senome* becomes a requirement. It is a necesssary intercessory instrument interposed between the cell and its environment. Thus, the *Senome* is the means by which cell responses, including epigenetic and genomic ones, coordinate in continuous confrontation with external environmental stresses.

It can be argued that there is justification in viewing the plasma membrane-based *Senome* as a kind of cellular “brain” [36]. When so considered, the behavioral complexity of archaea, bacteria and diverse unicellular eukaryotes can be explained [14,18–21,72,73] In like manner, the intelligence of syncytial plasmodia *Physarum polycephalum* [16,74–76], as well as the intelligence of plants and their robust communicative/cognitive faculties can be sufficiently understood [8,77–82]. Thus, the *Senome* provides a background through which the basal cognitive capacities of the cell, can be explored along pathways toward the type of consciousness that is typically ascribed to animals, and eventually to ourselves. The *Senome* can be seen as the link between qualia, as a unicellular form of sensorium, and higher levels of subjective consciousness as fundamental to the informational connections that ultimately apply to inherently cellular complex holobionic organisms. As the cellular interface between the outward environment and the living interior of cell, it can be further proposed that the *Senome* is at the express interface of physics and biology through which physical stimuli are translated into biological experiences that ultimately leads to human sentience (Box 1). As an explicit example, the *Senome* first generates electric signals at the plasma membrane and then transforms these electric signals into the chemical “language” of signaling networks impinging on the epigenome [56]. Only when contemporary biology will integrate the Genome, Epigenome and Senome altogether, will it be able to embrace the whole complexity of the eukaryotic cell to understand communicative assembly of cells in the form of multicellular organisms [43,46,48,49,51,68–71].

## Components of the Senome and their relevances for life

As proposed in this Opinion paper, the Senome is composed primarilly of a limiting plasma membrane (Box 1) which is the phospholipid double-layer equipped with myriad of proteins, lipids, sugars and other carbohydrates [83,84]. Characteristic features of the plasma membrane include its self-organization and inherent association with the actin cytoskeleton via its inner leaflet and diverse extracellular structures via its outer leaflet [83–89]. All life known on this planet is cellular. It is the assembly of lipid membranes that allowed the emergence of cellular life [90–92]. Membrane-based senes give all organisms their agency which is the central feature for their acting as genuinely living organisms. The totality of all senes is the *Senome* which underlies embodied cognition and sense of Self (see also the Glossary), allowing organisms to act in their own interest.

A very strong argument in a favour of the *Senome* for the still mysterious phenomenon of life is provided by viruses which are inert non-living structures as long as they are outside of their host cells [93]. However when inside their host cells and included within the senomic context, viruses can actively manipulate cellular membranes and the cytoskeleton. This allows them to form unique viral membraneous compartments, ultimately reaching access to the nucleus for both their replication and egress [94–97]. Similarly DNA acts as genetic blueprint of cells only if placed within the senomic context of cells whereas outside of cell, DNA is merely biologically inert macromolecule [68,98].

## Implications of the cellular Senome for multicellularity and social cognition

Finally, the Senome has potency to explain multicellularity based on cell-cell communication and cognition (Box 1) [68–71,99]. Social and emotional closeness, sympathy, bonding/friendship of humans are emergent self-similar aspects of shared subject *Senomes*, as seen for example in similar neural responses to sensory stimulation [49,100–103]. All this suggests that a shared species specific *Senome*, as collective information and communication, underlies social networks in humans [100,101]. Therefore, it is argued that the *Senome* has direct pertinence for understanding human perception and understanding of mental signals from outside (memes) or from within our own bodies (perceived as emotions and feelings) and further relevances for both individual and social cognition [100–107], that ultimately leads to the theory of mind [5,106,108,109] and human cultural evolution [110–113].

## Conclusions

The *Senome* translates physical signals from the outside world into the physico-chemical language of cells. It is central feature for all cellular life as it allows DNA and RNA to support life processes. Without membranes generating the Senome, DNA and RNA are rather inert macromolecule not able to support the living processes. We propose that the Senome is the inherent partner of the Genome and the Epigenome. The Senome underlies sensory order [114] which gives all cellular organisms a sense of time and agency [115]. Therefore, the Senome can be seen as the nexus between the prescribed cognitive capacities of the cell, its informational architecture and its working characteristics. All this require orderly regulation [115]. The Senome functions as a plasma membrane-based cell-wide sensory organ of assessment and action that permits continuous coordinated organismal-environmental complementarity. When placed in the context of cellular cognition, the Senome is that aspect of the cellular life that interrogates the environment and guides cellular responses and adaptation as a problem-solving phenomenon. The Senome should be added to our biological agenda as an additional means by which the entire complexity of the sentient and cognitive cell, and those crucial communicative aspects that lead to complex multicellular organisms, can be understood. Perhaps through this new exploration, based on inherent and primordial cell processes, a path towards the long-sought unification between physics and biology will be revealed.

## (Glossary)

### The Senome Concept in Short

**Sene**: Sensor-based sensory experience (event) which extract from environment information of adaptive value for the organism.

**Senome**: Sum of all sensory information of cell (or an organism) generated by the plasma membrane and its derived endosomal membranes. Sensors process and integrate sensory experiences into adaptive behavioural outputs. The Senome includes electric, magnetic and mechanic fields generated by (around as well as within) the organism – these then feed-back back into the epigenome and, finally, into the genome. At the multicellular level, Senome is represented by the sum of all synapses. Senome generates the Self – the subject – and drives its behaviour.

**Self**: Sum of all sensory information of an organism, integrated with information encoded both in the epigenome and genome, which allows generation of the organism subjective identity.

**Sentience**: Ability to perceive and feel environment subjectively.

**Subject**: Organism (agent) which has subjective sensory experiences generating its subjective consciousness, constructing its own world-view (active agency on, and relationship with, its environment).

**Organism (Agent**): Living system endowed with the Senome – Epigenome – Genome complex; capable of responses to stimuli, reproduction, growth and development, and maintenance of homeostasis. The organism is active in altering of physical environment to construct its own living niche as a problem-solving entity. It is capable of communication with, and manipulation of, other organisms sharing the same niche and environement.

**Genome**: Sum of all protein coding DNA/RNA-based hereditary information of an organism.

**Epigenome**: Sum of all non-coding DNA/RNA-based hereditary information of an organism. Besides the machinery regulating package of DNA into the chromatin and chromosomes, the epigenome includes also all structures acting as templates for their own copying. [149]
